# Improving Health Literacy for Individuals With Intellectual and Developmental Disability and Their Carers

**DOI:** 10.1192/bjo.2024.359

**Published:** 2024-08-01

**Authors:** Jesmine Dhooper, Mariam Omokanye

**Affiliations:** 1 University of Nottingham, Nottingham, United Kingdom; 2 Nottinghamshire Healthcare NHS Foundation Trust, Highbury Hospital, Nottingham, United Kingdom

## Abstract

**Aims:**

The aim of our project is to support health literacy in patients and carers under the Nottinghamshire Intellectual and Developmental Disabilities (IDD) service. To achieve this aim, we will produce a 20 page newsletter, containing updated and accessible research on mental health disorders that are common in the IDD population. We will also utilise a Trust webpage to publish the newsletter and produce a video/ podcast for the webpage, showcasing individuals with intellectual disabilities discussing and interacting with the articles.

**Methods:**

Research into the relevant articles included a search on Google Scholar and PubMed, and a list was written up. Final research articles to be included in the newsletter were selected after consultation with the consultant peer group within the Intellectual and Developmental Disability team within Nottinghamshire Healthcare NHS Foundation Trust. Easy read forms of all the articles were drafted by researchers, which will be sent to relevant authors to verify that this is an accurate representation of their research. An accessible 20-page newsletter will be produced, and an IDD focus group will review the content of the newsletter, discuss the articles and relevant videos/ podcasts will be made of these interactions. A webpage on the trust website will be created to publish the newsletter and allow users to interact with the articles electronically (using the standard electronic accessibility tools) and this will also contain the videos/podcasts produced. Feedback will be obtained electronically via a QR code and via traditional means e.g. an easy read reply slip.

**Results:**

The key outcomes of our project are producing 10 easy read articles within our newsletter. These articles need to be useful and accessible to the IDD population, which will be verified by small focus groups consisting of patients with IDD, carers and staff to review literature before publication as well as the feedback after publication. Another key outcome is the use of coproduction to involve people with IDD in production of the newsletter and webpage, in order to recognise the value of their lived experience, improve the quality of the project and drive success.

**Conclusion:**

Successful publication and feedback will pave a way for exploring a second edition the following year for printing via Trust communications. If successful, this project could be used as a template for an effective way to share research findings that contribute to the understanding of assessment and treatment pathways for people with an Intellectual and Developmental Disability.

